# A review of longitudinal clinical programs in US medical schools

**DOI:** 10.1080/10872981.2018.1444900

**Published:** 2018-03-15

**Authors:** Galina Gheihman, Tomi Jun, Grace J Young, Daniel Liebman, Krishan Sharma, Eileen Brandes, Barbara Ogur, David A. Hirsh

**Affiliations:** a Harvard Medical School, Boston, MA, USA; b Department of Medicine, Stanford University School of Medicine, Stanford, CA, USA; c Department of Medicine, Cambridge Health Alliance, Cambridge, MA, USA

**Keywords:** Undergraduate medical education, longitudinality, curriculum design, integration, continuity

## Abstract

**Background**: Longitudinal clinical experiences are a common component of undergraduate medical curricula, yet these programs have not been systematically characterized in US medical schools.

**Objective**: Our study sought to identify and characterize longitudinal clinical programs (LCPs) in US medical schools and measure associations between programs’ structures and goals.

**Design**: Using a mixed-methods approach, we conducted a secondary analysis of data from publicly available websites. We conducted a systematic keyword search of the websites of 137 LCME-accredited US medical schools to identify LCPs. We included programs with student–patient interactions of at least six months. We categorized programs using qualitative thematic analysis and compared associations between program structures and goals.

**Results**: We identified 98 LCPs in 69 schools. Half (52.0%) of LCPs occurred during the core clinical year. Program structures included ‘clinic attachments’ (50.0%), ‘longitudinal integrated clerkships’ (26.5%), and ‘patient attachments’ (20.4%). We identified goals in 89 programs, including ‘exposing students to specific topics, patient demographics, or practice settings’ (78.7%); ‘clinical or professional skill development’ (65.2%); and ‘understanding the patient experience’ (19.1%). Patient attachments were associated with ‘exposure to specific patient demographics’ (*P *= .04) and ‘understanding the patient experience’ (*P *= .03). Pre-clinical programs were associated with clinical skills development (*P *= .01).

**Conclusions**: Our study identifies the scope and nature of LCPs in US medical schools. Understanding connections between educational structures and goals may guide program design and research investigations of educational processes and outcomes.

## Introduction

Academic leaders in medicine have called for the transformation of health professions education [1–4]. The Lancet Commission Report [1] urges educational leaders to develop curricula that will serve patient and population needs, foster better understanding of the clinical context, emphasize continuous care over episodic encounters, and broaden training venues beyond inpatient care. To address these goals, medical schools are implementing curricular structures grounded in educational continuity [5–9], including longitudinal clinical programs (LCPs) [10–13] and a subset of LCPs, longitudinal integrated clerkships (LICs) [14–16].

In the LIC model, students care for a diverse panel of patients whom they follow across multiple clinical settings over extended periods of time, in lieu of traditional block rotations within discrete specialties [6,14,17]. The literature describes LIC goals, structures, and outcomes [15–21]; less is known about the broader array of LCPs that do not meet LIC criteria [10,13,22].

Worley et al. [23] describe a typology of LCPs, including both LIC and non-LIC programs, in seven countries. The authors subdivide 54 programs into three distinct clusters according to program length and discipline coverage [23]. This study highlights associations between program type and educational factors such as setting and faculty specialty. Another recent typology [22] characterizes LCPs in Canada, placing programs along a continuum of integration, continuity, and longitudinality.

Our study aimed to identify and characterize the full spectrum of LCPs in United States medical schools. We conducted a systematic search of all LCME-accredited US medical schools to generate a list of longitudinal student–patient programs. We used qualitative thematic analysis to determine the nature of the programs’ structures and goals, and we measured associations between them. To the best of our knowledge, this is the first study assessing the landscape of LCPs in the US and associations between LCP structures and goals.

## Methods

### Study design

This mixed-methods study was a secondary analysis of existing data from publically available medical-school websites and other media.

### Online search strategy

We performed an online search using the search function on each medical school’s website and seven key phrases: longitudinal patient, longitudinal experience, longitudinal clinical, longitudinal clerkship, longitudinal rotation, longitudinal relationship, and integrated clerkship. The search included all LCME-accredited US medical schools as of August 2014 (*n* = 137). Additionally, we performed a search of the schools’ names in conjunction with each of the key phrases using an independent search engine (Google, Mountain View, CA, USA). All searches were conducted between 24 August 2014 and 24 October 2014. We derived and documented details on the programs from the search results and sent a follow-up email to a contact person from each program or school to confirm program details.

### Longitudinal clinical program (LCP) eligibility

We included LCPs that were (1) designed for undergraduate medical students, (2) were at least six months in length, (3) had a stated emphasis on longitudinality, and (4) offered continuity with patients. We limited programs to those at least six months in length based on the determination of ‘longitudinal’ in a narrative review [19]. Rather than relying solely on program length, we required a *stated* emphasis on longitudinality as a proxy for schools’ deliberate intentions to create curricular connectivity [6]. We defined ‘continuity with patients’ as the opportunity for a student to meet with a given patient more than once during the program. When continuity with patients was not explicitly stated or confirmed by program administrators, at least two authors had to conclude independently, based on the available information, that continuity with patients was possible within the program’s design. We excluded programs if their design precluded continuity with patients or if there was insufficient evidence of continuity with patients. We defined ‘clinical’ programs as programs that fostered student–patient relationships, independent of whether the program took place in the clinical or the pre-clinical years of medical school.

### Data extraction

We extracted program characteristics and the explicitly-stated program goals from program websites or from other online sources describing the program, such as publically available news articles. Based on domains examined in an earlier review of longitudinal community and hospital placements [13], we extracted the following six structural characteristics for each LCP: date established, curricular vs. extracurricular, mandatory vs. elective, phase of training, program length, and program type (LIC, patient attachment, clinic attachment, or both patient and clinic attachment). We classified programs as LICs based on self-identification or by applying the 2007 consensus definition [15,16,18,19]. We defined ‘patient attachments’ as programs where students selected or were assigned patients to follow over time, either within a single venue or across multiple clinical sites. We defined ‘clinic attachments’ as programs where students were assigned to a specific preceptor or clinical site and saw patients in that venue over time.

When programs met criteria for patient attachment and clinic attachment but not the full LIC definition (specifically, *not* offering ‘the majority of the academic year’s core clinical competencies across multiple disciplines simultaneously’) [15,16,19], we characterized these as ‘both patient and clinic attachments’ (see Supplementary Material 1, which depicts and characterizes types of LCP programs—patient attachment, clinic attachment, or both patient and clinic attachment). Because patient and clinic attachments may not be mutually exclusive (i.e., students assigned to one clinic would likely see the same patients over time), we made definitions based on the programs’ described structures and not on the students’ experiences of these structures.

### Data analysis

#### Qualitative

Six investigators (TJ, GG, GY, DL, KS, and EB) coded data using thematic analysis [24]. The programs were divided into three groups with two investigators assigned to review each group. Initially, two investigators independently reviewed all programs in their assigned group and identified recurrent patterns among the extracted goals. All six investigators then developed an empiric coding framework together following an iterative, consensus-building process [25]. Next, the same teams of two investigators independently assigned codes to the extracted goals for each program within their group and reconciled differences through discussion. Three rounds of this process led to the final set of codes and their assignment to each program. Investigators assigned codes based on a literal interpretation of the stated program goals. Through an iterative consensus building process, the six investigators clustered minor codes into major categories based on common themes. One investigator (TJ) reviewed all final code assignments and converted codes to counts for statistical analysis.

#### Quantitative

We compared the characteristics of programs’ structures to program goals arising from the qualitative analysis. We evaluated relationships by using the Chi-square test for independence (Excel 2010, Microsoft, Seattle, WA, USA). For any category with five or fewer programs, we used the Fisher exact test for small numbers (Richard Lowry, http://vassarstats.net/fisher2x3.html). The significance threshold was set at .05.

We compared four of the six extracted structural characteristics (mandatory vs. elective, program phase of training, program length, and program type) to seven selected program goals to determine associations. We did not use the program characteristic ‘curricular vs. extracurricular’ for comparison as only three programs were extracurricular. We did not compare goals by ‘date of establishment’ because a specific date could not be determined in nearly a third of the programs. We selected the seven program goals for the quantitative association analysis from among codes derived emergently in the qualitative analysis. We chose these goals for the association analysis without prior review of the quantitative data. We selected the seven program goals by considering themes currently prominent in the clinical medical education design literature, including educational continuity [5–8,21,26], longitudinal programs [13,23], LICs [15,16,18,27,28], and the future of medical education in the US [1–4]. The seven program goals selected were ‘exposure to specific patient populations,’ ‘exposure to social medicine,’ ‘exposure to primary care,’ ‘developing clinical skills,’ ‘developing professional skills,’ ‘understanding the patient experience,’ and ‘fostering longitudinal relationships.’

## Results

The search of the 137 LCME-accredited US medical schools yielded 98 LCPs meeting our inclusion criteria in 69 medical schools. Our follow-up emails to program administrators yielded responses from 53% of programs.

### Characteristics of longitudinal clinical program structure

Characteristics of the structure of the 98 LCPs are summarized in Table 1. Twelve programs (12/98, 12.2%) were from six months to less than one year in length, and forty-seven programs (47/98, 48.0%) were one year in length. Thirty-nine programs were greater than one year in length (39/98, 39.8%). Forty-six programs (46/98, 47.0%) were mandatory parts of the curriculum. Fifty-one programs (51/98, 52.0%) took place exclusively in the clinical years, 26 (26/98, 26.5%) took place exclusively in the pre-clinical years, and 21 (21/98, 21.4%) extended through both the pre-clinical and clinical years of medical school. Twenty schools offered more than one distinct LCP. The most common program type was the clinic attachment (49/98, 50.0%). The remainder were LICs (26/98, 26.5%), patient attachments (20/98, 20.4%) and programs that had both clinic and patient attachment components (3/98, 3.1%) (See Supplementary Figure 1 for an overview of the program types).

**Table 1.** Characteristics of longitudinal clinical program structure among included programs at US medical schools.

Program characteristics	Number (%)
**Total schools searched (*n* = 137)**	
Schools with one longitudinal clinical program identified	49 (35.8)
Schools with two or more longitudinal clinical programs identified	20 (14.6)
Schools with no longitudinal clinical programs identified	68 (49.6)
**Total programs identified (*n* = 98)**	
Programs with stated goals	89 (90.8)
Programs with no stated goals	9 (9.2)
**Date established (*n* = 98)**	
Prior to 2000	7 (7.1)
2000 to 2004	12 (12.2)
2005 to 2009	12 (12.2)
2010 to 2014	37 (37.8)
Unknown	30 (30.6)
**Curricular vs. extracurricular (*n* = 98)**	
Curricular programs	95 (96.9)
Extracurricular programs	3 (3.1)
**Mandatory vs. elective (*n* = 98)**	
Mandatory (all students participate)	46 (46.9)
Elective (not all students participate)	52 (53.1)
**Phase of training (*n* = 98)**	
Programs exclusively in pre-clinical years	26 (26.5)
Programs exclusively in clinical years	51 (52.0)
Programs spanning pre-clinical and clinical years	21 (21.4)
**Program length (*n* = 98)**	
6 months to <1 year	12 (12.2)
1 year	47 (48.0)
>1 year	39 (39.8)
**Program type (*n* = 98)**	
Longitudinal integrated clerkship (LIC)	26 (26.5)
Patient attachment only	20 (20.4)
Clinic attachment only	49 (50.0)
Patient and clinic attachment	3 (3.1)

### Prevalence of stated programmatic goals

Of 98 LCPs identified, 89 programs (89/98, 90.8%) were found to have explicitly stated goals. The number of goals per program ranged from 1 to 16 (mean = 3.72 goals). We derived 39 codes from these goals and organized the codes into major, minor, and sub-categories. Table 2 demonstrates the number of programs corresponding to each code.

**Table 2.** Prevalence of the major, minor, and sub-categories of stated program goals among included Longitudinal Clinical Programs at US medical schools.

Program goals	Number/total programs (%)
**Exposure^a^**	70/89 (78.7)
*Specific patient demographics^b^*	33/89 (37.1)
Geriatric patients^c^	5/89 (5.6)
Underserved patients	8/89 (9.0)
Urban patients	4/89 (4.5)
Rural patients	9/89 (10.1)
Patients with chronic disease	9/89 (10.1)
Other	5/89 (5.6)
*Specialties*	22/89 (24.7)
Primary care	21/89 (23.6)
Other	1/89 (1.1)
*Outpatient/Community-based care*	15/89 (16.9)
*Social Medicine*	36/89 (40.4)
Social determinants of health	16/89 (18.0)
Psychosocial aspects of health	12/89 (13.5)
Healthcare system	22/89 (24.7)
**Recruitment**	10/89 (11.2)
Specific geographic area	7/89 (7.9)
Specific specialty	5/89 (5.6)
**Skill development**	58/89 (65.2)
*Clinical Skills*	39/89 (43.8)
History and physical	12/89 (13.5)
Clinical reasoning and diagnosis	9/89 (10.1)
Plan and management	7/89 (7.9)
Communication with patients	9/89 (10.1)
Prevention/health maintenance	6/89 (6.7)
Shared decision making	2/89 (2.2)
Patient-centered care	8/89 (9.0)
Unspecified	17/89 (19.1)
Other	3/89 (3.4)
*Professional Skills*	39/89 (43.8)
Professionalism	10/89 (11.2)
Humanism	10/89 (11.2)
Ethics	6/89 (6.7)
Leadership	5/89 (5.6)
Cultural competency	13/89 (14.6)
Interprofessional team-based care	13/89 (14.6)
Advocacy	2/89 (2.2)
Lifelong learning	4/89 (4.5)
Quality improvement	4/89 (4.5)
Other	4/89 (4.5)
**Understanding the patient experience**	17/89 (19.1)
**Foster longitudinal relationships**	29/89 (32.6)
Between student and supervisor	7/89 (7.9)
Between student and patient	24/89 (27.0)
Between student and clinical team	1/89 (1.1)
Between student and other	3/89 (3.4)
**Integrate pre-clinical and clinical curricula**	10/89 (11.2)

^a^Six major categories of stated programmatic goals were included in the framework and are indicated in **bold** above. An LCP could be assigned one or more of the codes under this category.

^b^Minor categories were identified as sub-groups within certain major categories and are indicated in *italics* above. An LCP could be assigned one or more of the codes under this sub-group.

^c^Sub-categories were further subdivided within certain minor categories and are identified in plain text above. A program could be assigned one or more of the codes under this sub-group.

Providing students with exposure to specific topics or practice settings was the most common major programmatic goal (70/89, 78.7%). For example, 40.4% of programs (36/89) emphasized exposure to social medicine including the social determinants of health, psychosocial aspects of health, and engaging with the healthcare system. Exposing students to specific patient demographics, such as geriatric patients, rural patients, and individuals with chronic disease, was a stated goal for 37.1% of programs (33/89).

Fifty-eight programs (58/89, 65.2%) had programmatic goals highlighting skill development. The minor categories within this major category were ‘developing clinical skills’ and ‘developing professional skills’. These domains overlapped; 20 programs (20/89, 22.5%) listed both clinical and professional skills as goals, 19 programs (19/89, 21.3%) listed only clinical skills, and 19 (19/89, 21.3%) listed only professional skills.

Twenty-nine programs (29/89, 32.6%) stated goals related to fostering longitudinal relationships. Twenty-four of these programs (24/89, 27.0%) explicitly listed student–patient relationships as a goal, and seven programs (7/89, 7.9%) emphasized the relationship between student and supervisor. Other programmatic goals included understanding the patient experience (17/89, 19.1%), integrating pre-clinical and clinical curricula (10/89, 11.2%), and recruiting graduates to a specific geographical location or specialty (10/89, 11.2%).

### Associations between longitudinal clinical program structure and goals

**Figure 1.**
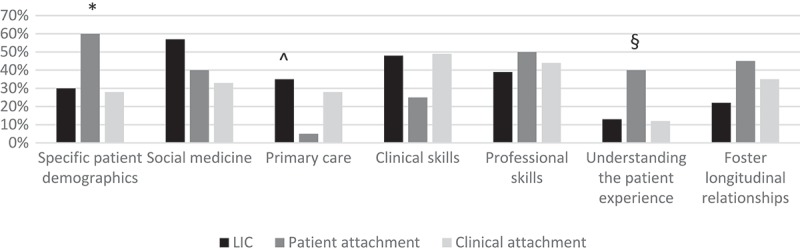
Longitudinal clinical program type (LIC, patient attachment, or clinic attachment) is associated with programmatic goals including ‘exposure to specific patient demographics,’ ‘exposure to primary care,’ and ‘understanding the patient experience’. *Association between patient attachment and “exposure to specific patient demongraphics” (P=0.04) ˆAssociation between LIC and “exposure to primary care” (P=0.04) §Association between patient attachment and “understanding the patient experience” (P=0.03)

We found statistically significant associations between characteristics of LCP structure and stated goals. Program type (LIC, patient attachment, or clinic attachment) was associated with the goals ‘exposure to specific patient demographics’ (*P *= .04), ‘understanding the patient experience’ (*P *= .03), and ‘exposure to primary care’ (*P *= .04) (Figure 1).

When we compared program types directly, we found patient attachment programs were more likely to have goals related to exposing students to specific patient demographics than either LICs (*P *= .02) or clinic attachments (*P *= .01). Patient attachment programs were also more likely to emphasize understanding the patient experience than clinic attachments (*P *= .02), although there was no significant difference between patient attachments and LICs in this regard (*P *= .07). Patient attachments were less likely than either LICs (*P *= .02) or clinic attachments (*P *= .05) to emphasize exposure to primary care.

**Figure 2.**
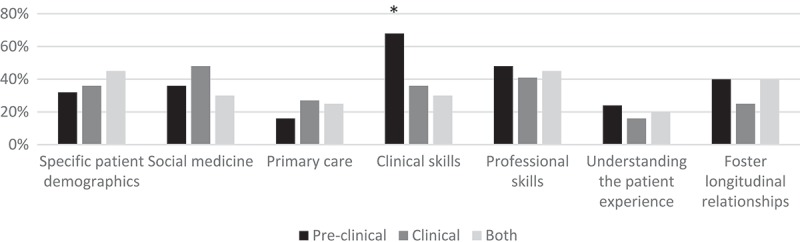
Longitudinal clinical program phase of training (Pre-clinical, clinical, or both) is associated with the programmatic goal of ‘developing clinical skills.’ *Association between pre-clinical phase of training and “developing clinical skills” (P=0.01)

Phase of training (pre-clinical, clinical, or both) was associated with the goal ‘developing clinical skills’ (*P *= .01) (Figure 2). When compared directly, pre-clinical programs were more likely than either programs exclusively in the clinical phase of training (*P *= .01) or programs spanning both phases of training (*P *= .01) to emphasize clinical skills as programmatic goals.

We found no relationships between stated goals and program length or whether the program was mandatory vs. elective.

## Discussion

Our online search of all LCME-accredited US medical schools in 2014 identified 98 LCPs meeting our inclusion criteria at 69 medical schools and demonstrated associations between program structures and programmatic goals. This study examined LCPs that were at least six months in length, were designed for undergraduate medical students, had an emphasis on longitudinality, and offered continuity with patients. At least half of US medical schools offered LCPs meeting our criteria. Twenty schools in the US offer more than one distinct LCP. Programs differed in structure, including date established, curricular vs. extracurricular, mandatory vs. elective, phase of training, program length, and program type (LIC, patient attachment, clinic attachment, or both patient and clinic attachment).

We found associations between characteristics of program structure and goals that imply a degree of coherence across LCPs. We identified a significant association between LCPs in the preclinical years and the stated goal of developing clinical skills (*P *= .01). Dornan et al. (2006) systematically reviewed medical students’ experiences in the ‘pre-clinical phase’ and highlighted multiple goals we found (social determinants of health, psychosocial aspects of health, understanding the healthcare system, clinical skill development, and professional skill development); however, other than ‘clinical skills,’ our data did not significantly associate these other goals with the pre-clinical phase. We speculate that pre-clinical LCPs may recruit students by offering opportunities to practice clinical skills prior to beginning the core clinical year [29]. It is also possible that this finding heralds the longstanding concern about inadequate explicit emphasis on clinical skill training in the core clinical years [30].

We found other structures that connected to program goals. ‘Patient attachment’ programs were more likely than other program types to emphasize specific patient demographics (*P *= .04) and support the goal of ‘understanding the patient experience’ (*P *= .03). This association is perhaps unsurprising, as ‘patient attachments’ by definition emphasize pairing students with individuals from particular patient populations, independent of the clinic site or mentor. This finding is consistent with studies of senior mentor programs—a type of patient attachment—which found such programs could improve students’ understanding of and attitudes towards older patients [31,32].

On the other hand, our finding that patient attachment programs were more likely than LICs (*P *= .02) to have goals related to exposing students to specific patient demographics may mark a difference between US LICs and Canadian and Australian LICs. The literature documents LICs in Canada [33] and in Australia [34] are often created for the explicit goal of serving Aboriginal and other underserved communities [12,15,16]. Of course, some medical schools in the US have stated goals of serving rural populations [11,35–38], but the discrepancy found in our analysis suggests that our study may not have recognized some LIC programs designed to serve demographic groups defined by race, ethnicity, or place (such as rural and remote populations). Our finding also may invite questions about the degree to which social accountability informs educational design of the core clinical year in the US [39].

In our study, 73.5% of programs offered longitudinal structures with LIC-like elements but did not meet the criterion of the international research definition of an LIC that states LICs must ‘meet the majority of the academic year’s core clinical competencies across multiple disciplines simultaneously’ [15,16,18,19]. By using a broader definition of LCPs, our study characterizes longitudinal programs which would have been excluded under stricter criteria. Hereby, we found that both the non-LIC clinic attachments (50.0% of LCPs in our study) and LICs (26.5% of LCPs) name primary care exposure and recruitment as explicit goals significantly more often than non-LIC longitudinal patient attachment programs (*P *= .05 and *P *= .02 respectively). These findings connect to outcomes in the literature demonstrating that LICs effectively support the primary care workforce [11,19,40,41]. We also consider these findings in the context of earlier studies demonstrating that 95% of students seeking primary care careers ‘desire for longitudinal patient care opportunities’ [42, p. 324]. Whether non-LIC LCPs such as clinic or patient attachments recruit and retain for primary care workforce remains unclear [10,13,23].

Our work builds upon earlier studies that review LCPs and LCP-like programs. Thistlethwaite et al. (2013) systematically reviewed longitudinal placements and highlight program outcomes that connected to program goals featured in our data, notably ‘the development of a patient-centred approach to clinical care, presentations of illness in primary care settings and the development of insights into community-based medicine’ [13, p. e1352]. The authors describe the importance of educational continuity [6,15,16], a framework based in creating longitudinal relationships, and a goal found among 29/89 (32.6%) of US medical schools in our study. Although the authors’ outcomes data appear to support the goals we discerned among US LCPs, it is noteworthy that their systematic review included programs 13 weeks or more in length whereas our review—consistent with two earlier works [19,43]––only included longitudinal programs six months in length or longer.

The differences in the lengths of the programs these studies included, and variations in length among US programs in our study, raise the question of how much time is needed to define a program as ‘longitudinal.’ Two papers begin to address the question of defining longitudinality by suggesting typologies of longitudinal programs. Worley et al. (2016) identified 54 LCPs across seven countries and categorized them according to program length and discipline coverage [23]. Worley et al. (2016) identified three major clusters of LCPs: programs that fit the traditional definition of LICs (‘Comprehensive LICs’), programs that blended the LIC format and traditional block structure for equal parts of the year (‘Blended LICs’), and LCPs that offered integration of some disciplines over time, but with a longitudinal design of less than half a year (‘Amalgamative Clerkships’) [23]. Our results extend the typology of Worley et al. (2016) [23], which only characterized programs occurring in the core clinical year, by including LCPs in all years of medical school. In other ways, our study was more restrictive than the survey by Worley et al. (2016); we included programs of six months’ duration or more, and therefore, the Amalgamative Clerkships described by Worley et al. (2016) would have been excluded from our study [23].

Ellaway et al. (2016) characterized LCPs in Canada by undertaking a study with theoretical and empirical dimensions [22]. Based on their results, Ellaway et al. (2016) offer a typology that organizes programs along a continuum ‘based on the extent to which integration, continuity and longitudinality are expressed’ [22, p. 918]. This typology highlights a way to assess longitudinal clinical education in programs without relying on a strictly length-based definition [13,23]. Through this lens, we see the diversity of programs that use continuity as an organizing principle to foster longitudinal relationships among students, patients, and providers [6,19,36,43].

### Limitations

Although our study included all LCME-accredited US medical schools, our search strategy relied upon publicly available information from schools’ websites; therefore, our search may be out of date, may misrepresent the true prevalence of LCPs, or may not fully depict schools’ current LCPs’ structures and goals. We used seven specific keywords in our systematic search; it is possible that other search terms may have yielded more LCPs meeting our criteria. To improve accuracy, we supplemented our search by contacting program administrators, and 53% of programs responded; yet, missing data or errors may persist among the non-respondents. In addition, eligible programs may have been excluded because schools may not post information about all current programs on their websites. Although we followed standard qualitative research methods for coding, our coding was limited by the quality of the information available. We limited our quantitative analysis to seven program goals (related to themes prominent in the literature) in order to identify significant associations while limiting false positives that may occur with multiple comparisons. Our approach may have missed other significant associations between program structures and goals that we did not examine. Finally, this study only included allopathic medical schools; we do not know the extent of LCPs in osteopathic medical schools or their structures and goals.

## Conclusion

We have identified LCPs in US medical schools and described associations between LCP structures and programmatic goals. To our knowledge, our study is the first to characterize LCPs (both LICs and non-LICs) in the US. In addition, our literature review found no other studies that characterized LCPs of at least six months’ duration unlimited by the year in the curriculum.

Future research is needed to determine how program structures can support educational, institutional, and workforce outcomes. Ultimately, clinical education leaders should continue to innovate and study programmatic structures to create the outcomes that learners, patients, and society need most.
