# Supplementary material for: A review of longitudinal clinical programs in US medical schools
Source: Med Educ Online. 2018 Mar 15;23(1):1444900. doi: 10.1080/10872981.2018.1444900 (PMC5907349; doi:10.1080/10872981.2018.1444900)
Supplement: Supplementary_Material_1_Nov_5.docx [file ZMEO_A_1444900_SM6377.docx]

**Appendix 1: Longitudinal Clinical Program Types**

Longitudinal clinical programs (LCPs) were categorized as patient attachments, clinic attachments, both clinic and patient attachment programs, or longitudinal integrated clerkships (LICs). Each program type is illustrated schematically, followed by a definition and example.


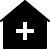

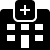

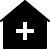


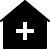

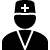

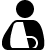


Orthopedics

Clinic

Orthopedics

Clinic

Primary Care

Center

Rehabilitation

Hospital

**Patient Attachment**

*Definition: Involves the regular, recurrent relationship with the same patient (or several patients) over time across multiple different clinical settings. The student encounters different clinical preceptors in different settings or there may be no clinical preceptor during an encounter (e.g. during a home visit or telephone call with the patient).*

Example: A student meets Mr. J in orthopedics clinic. The student follows Mr. J’s clinical course over time and across settings, joining him at a rehabilitation therapy appointment, a primary care appointment, and a follow-up appointment in the orthopedics clinic.


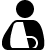

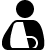

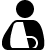

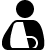

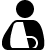

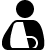


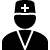

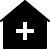


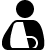

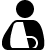

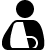

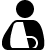


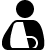


**Clinic Attachment**

*Definition: Involves the regular, recurrent placement in the same clinical setting with the same preceptor over time (and thus an opportunity to access the same patient base for longitudinal follow-up).*

Example: A student is assigned to a longitudinal placement in a neurology clinic. With the neurology preceptor, the student meets several patients (Mr. A, Mr. B, Ms. C, and Ms. D); some of these patients (Mr. A, Ms. D) the student will see on multiple occasions in that neurology clinic for longitudinal follow-up.


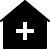

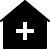


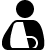

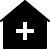

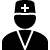


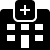


**Clinic and Patient Attachment**

*Definition: Involves the regular, recurrent placement in the same clinical setting with the same preceptor over time (and thus an opportunity to access the same patient base for longitudinal follow-up). Additionally, the student is assigned specific clinic patients to build a longitudinal relationship with, and may follow these patients both at the clinic and across multiple clinical settings. However, unlike longitudinal integrated clerkships, the student will not fulfill the majority of the year’s clinical competencies in this program.*

Example: A student is assigned to a longitudinal placement in a cardiology clinic. They meet several patients whom they see on multiple occasions during longitudinal follow-up in cardiology. The student also meets a patient Ms. K in cardiology clinic whom the student joins at a primary care appointment and a pulmonology clinic follow-up.

**Longitudinal Integrated Clerkships (LICs)**

*Definition: An LIC is characterized by being the central element of clinical education whereby medical students: (1) participate in the comprehensive care of patients over time, (2) participate in continuing learning relationships with these patients’ clinicians, and (3) meet the majority of the year’s core clinical competencies, across multiple disciplines simultaneously through these experiences. In this way, LICs resemble programs that are both clinic and patient attachments (with students assigned to regular, recurrent placements in several clinical settings), yet differ from the above in that students are assigned to participate in several clinic and patient attachments across multiple disciples simultaneously for the majority of the clinical year.*

Example: A student undertakes all of their core clinical year by caring for cohorts of patients working longitudinally with their preceptors in clinics. The cohorts of patients are cared for with faculty preceptors spanning the core required disciplines determined by the school—e.g. the student works year long simultaneously in medicine, surgery, obstetrics/gynecology, pediatrics, psychiatry, and neurology, alongside serial engagements with their patients and other patients in the operating room, emergency room, and radiology.

**Icon References:**

Physician: <https://icons8.com/web-app/9570/Medical-Doctor-Filled>

Patient: <https://icons8.com/web-app/9531/Triangular-Bandage-Filled>

Clinic: <https://icons8.com/web-app/1782/clinic#filled>

Hospital: <https://icons8.com/web-app/9508/Hospital-3-Filled>
